# Supplementary material for: Open problems in ageing science: a roadmap for biogerontology
Source: GeroScience. 2025 Nov 8;48(3):3351–60. doi: 10.1007/s11357-025-01964-4 (PMC13356218; doi:10.1007/s11357-025-01964-4)
Supplement: Supplementary file 1 — (DOCX 19.6 KB) [file 11357_2025_1964_MOESM1_ESM.docx]

**Supplementary material**

Talay et al., *Open problems in ageing science: A roadmap for biogerontology*

| **Metric** | **All Problems** | **Top 20 Problems** | **Bottom 20 Problems** |
| --- | --- | --- | --- |
| **Total Articles** | 172,031 | 69,322 | 341 |
| **Mean Articles per Problem** | 847.44 | 3,466.1 | 17.05 |
| **Median Articles per Problem** | 437.0 | 2,840.5 | 17.5 |
| **Range of Articles** | 1 to 10,808 | 2,196 to 10,808 | 1 to 36 |

**Supplementary Table 1:** Summary statistics of all open problems and the counts of relevant PubMed articles found to be relevant for the open problem.

**Supplementary Table 2:** Final list of 100 open problems, available as a separate PDF file.
