# Supplementary material for: Open problems in ageing science: a roadmap for biogerontology
Source: GeroScience. 2025 Nov 8;48(3):3351–60. doi: 10.1007/s11357-025-01964-4 (PMC13356218; doi:10.1007/s11357-025-01964-4)
Supplement: Supplementary file 2 — (PDF 98.5 KB) [file 11357_2025_1964_MOESM2_ESM.pdf]

Supplementary Table 2: Final List of 100 Open Problems

Ageing Mechanisms
What are the causal mechanisms of human ageing?
Is ageing a collection of independently developing age-related diseases or is it mediated by common underlying processes?
Which tissues, organs or cell types contribute more to ageing?
How does the body compensate and adapt to any age-related change?
Do cell-intrinsic events (DNA damage, proteotoxic stress, mitochondrial dysfunction etc) drive ageing, or is ageing driven by systemic factors (hormones, immune system) and/or tissue microenvironment (extracellular matrix, cell-cell communication, stem cell niche)?
How much do hormonal (e.g., estrogen, testosterone, thyroxine, growth hormone, IGF1) changes contribute to ageing?
What is the contribution of different mechanisms/hallmarks to different ageing phenotypes and diseases?
How much does the involution of thymus contribute to ageing?
What genes and pathways must be targeted to extend lifespan and healthspan and how can we identify them?
How much does the continuation of developmental programs contribute to ageing?
What is the optimal level of autophagy in each tissue to optimize longevity?
What molecular and cellular processes modulate the pace of ageing in mammals?
How much do somatic mutations in promoter and regulatory regions contribute to ageing?
Which resilience/protective factors allow us to reach our current lifespan?
How much do positive feedback loops and chain reactions contribute to ageing?
How much does deregulation of gene expression programs, from epigenetic changes/drift or transcription factors, contribute to ageing?

Ageing and Disease
How many common triggers are there for age-related diseases?
Do fundamental ageing processes exist and if so, how do they synchronize ageing changes and promote age-related diseases?

How do ageing changes and processes influence susceptibility (and in some cases resistance) to diseases?

Biomarkers and Measurement
Are biomarkers of ageing accurately measuring ageing processes or rather measuring health?
How can we develop methodologies capable of quantifying different aspects of ageing, such as oxidation levels, senescent cells and DNA damage, in human tissues?
How can we measure intrinsic biological age in individuals and translate this knowledge into accurate biomarkers of ageing?
Can we come up with better markers that represent ageing changes in mice that are measurable in human samples (not restricted to blood)?
What do the current (epigenetic) clocks measure, i.e. which cellular/molecular processes do they reflect?
What does the minimal dataset look like that we would need to study the different aspects/trajectories of ageing?
To what extent are causes of natural variation in ageing rates modulated by environment and gene-by-environmental interactions?
How can we evaluate and compare epigenetic clocks within humans and with other species?
How can we measure the extent and pace of changes in the homeodynamic space during ageing?

Cellular Process
What is the role of senescent cells in normal physiology?
How much do stem cell exhaustion and dysfunction contribute to ageing?
How much does cell depletion contribute to ageing?

How much does the SASP contribute to ageing and disease?
How much do paracrine factors contribute to ageing?
Which and when are senescent cells beneficial or detrimental?
How does the regulation of biological time transitions across the life cycle occur?
Can we separate epigenetic cellular rejuvenation from reversal of cellular fate?
How much does excessive cell death and cell cycle arrest as well as suboptimal cell proliferation and renewal contribute to ageing?

Diversity in Human Ageing
Why does ageing manifests differently in humans, even close relatives?
What is the effect of sexual dimorphism and ethnicity on different aspects of ageing?
What are the genetic causes (genes and DNA variants) that contribute to natural variation in ageing rates?

Environmental and Physical Factors
How much does ageing increase susceptibility to environmental death causes?
How much do environmental heat and cold contribute to ageing?

Evolution and Comparative Biology
What mechanisms determine the longevity of long-lived species?
How much of ageing is an evolutionary adaptation?
Is ageing required to accelerate species evolution?
How many individual animals survive long enough in the wild to experience ageing?
Are there common biological patterns of ageing shared by birds and mammals?

How can the multifactorial origins of late-life disease be reduced to a small number of general biological principles, drawing on the evolutionary theory of ageing?
Can concepts from programmatic theory describe the origins and mechanisms of emergence of diseases of ageing?
How does antagonistic pleiotropy contribute to age-related diseases?
How do we find the major common denominators of ageing across the tree of life?
What are the mechanisms that lead to major changes in longevity, especially in mammals such human beings that live almost twice as long as chimps and age much slower than mice or rats?
How much does economical distribution of resources (e.g. to reproduction, fight or flight, or hibernation, instead of reparation) contribute to ageing?
Which gene variants exhibit antagonistic pleiotropy? Can we find variants that improve fitness early in life but are detrimental (e.g. increase mortality) late in life?

Immune System and Inflammation
Can targeting inflammation in older age slow down the process of tissue ageing?
How much does immunosenescence contribute to ageing?
To what extent does inflammageing and autoimmunity contribute to organismal ageing?
How much does immune cell clonal expansion contribute to ageing?
How much does the immune response to mutated cells or altered (oxidized, misfolded) molecules (e.g. oxLDL, beta amyloid, alpha synuclein) contribute to ageing?
How does the immune response to intracellular and extracellular pathogens contribute to ageing?

Interventions
How do life-extending interventions work and are they targeting similar or different processes?
How can we reverse or restore cell function lost during ageing?
Can a drug slow human ageing and how can we prove it?
How and which interventions should be prioritized for human clinical trials?
What is the best age, timing, duration, and dosage for various anti-ageing treatments?
Can an organ transplant from a long-lived animal extend lifespan in a shorter-lived species?
What is the basis for sex and genotype-specific differences in response to different anti-ageing interventions?
Which longevity interventions retard ageing and which modulate specific diseases like cancer?
Does partial reprogramming retard ageing in vivo?
How conserved across species are longevity interventions?
Can we apply stem cell therapy based on our own young stem cells (e.g., from cord blood) to modulate ageing?
How many organs in an old individual do we need to replace with young organs in order to reverse ageing?
How can we break through the current human lifespan ceiling of 122 years?
Can senolytics and consequent compensative replication for replacing the removed senescent cells accelerate telomere shortening and/or increase replication errors?
Which ageing changes and pathologies can and which cannot be reversed by cellular rejuvenation?

Could blood clean-up be used to target ageing processes?
Can we harness processes of embryogenesis to develop rejuvenation therapies?
Can we develop screens for anti-ageing efficacy that will work in people (and mice), but whose effects do not require 5 - 40 years to measure in people?
Will therapies based on young cells work in an aged niche/environment, what is the contribution of the systemic environment to ageing?
How can we develop pharmaceutical agents and interventions to target and reverse epigenetic modifications across different cell types to address age-related degeneration?
Can a combination of senolytics with ROCK inhibitors and 5-LOX inhibitors contribute to tissue rejuvenation?
How can we test whether longevity drugs given to mice make them younger (as opposed to improving health)?

Model Organisms
Which mechanisms are essential for ageing across a wide variety of model organisms, and which are unique to each model organism?
Which ageing changes in model organisms also change in a similar way in humans?
Why do genetically identical organisms (e.g. C57BL/6 mice) kept in identical environment still have roughly 2 fold variability in their lifespans?

Molecular Mechanisms
How much does telomere shortening contribute to ageing?

How much does the accumulation of malfunctioning cells contribute to ageing?
Does somatic mutation accumulation cause ageing or can it be an adaptive response to ageing-stress?
How much do errors in biochemical synthesis of metabolites contribute to ageing?
How much do errors in DNA replication contribute to ageing?
How much does suboptimal efficiency of DNA repair systems contribute to ageing?
How much does ferroptosis contribute to ageing?
How much does the accumulation of intracellular and extracellular waste contribute to ageing?
To what extent does the transformation of the extracellular matrix during ageing affect the functionality of various tissues?
What is the influence of chaperone translation and post-translational modifications on ageing, and to what degree does chaperone function diminish with age?
How many diseases of ageing are caused by the trapping of citrate in the mitochondria?
How much does suboptimal telomerase activity contribute to ageing?
How much does accumulating damage to the DNA contribute to ageing and which types of DNA damage are more important?
How much does the DNA damage response contribute to ageing?
Are there additional solute carrier (SLC) membrane transport proteins known to play roles associated with ageing?
